# Supplementary material for: New Approaches to Manage Asian Soybean Rust (Phakopsora pachyrhizi) Using Trichoderma spp. or Their Antifungal Secondary Metabolites
Source: Metabolites. 2022 Jun 1;12(6):507. doi: 10.3390/metabo12060507 (PMC9227527; doi:10.3390/metabo12060507)
Supplement: Supplementary file 1 [file metabolites-12-00507-s001.zip › metabolites-1707467-SI.pdf]

Article

New approaches to manage Asian soybean rust (*Phakopsora pachyrhizi*) using *Trichoderma* spp. or their antifungal secondary metabolites

Abbas El-Hasan[1,*], Frank Walker[2], Iris Klaiber[3], Jochen Schöne[2], Jens Pfannstiel[3] and Ralf T. Voegele[1,*]

1 Department of Phytopathology, Institute of Phytomedicine, Faculty of Agricultural Sciences, University of Hohenheim, Otto-Sander-Str. 5, D-70599 Stuttgart, Germany
2 Central Chemical-Analytical Laboratory, Institute of Phytomedicine, Faculty of Agricultural Sciences, University of Hohenheim, Otto-Sander-Str. 5, D-70599 Stuttgart, Germany; frank.walker@uni-hohenheim.de (F.W.), jochen.schoene@uni-hohenheim.de (J.S.)
3 Core Facility Hohenheim, Mass Spectrometry Unit, University of Hohenheim, Ottilie-Zeller-Weg 2, D-70599 Stuttgart, Germany; iris.klaiber@uni-hohenheim.de (I.K.), Jens.Pfannstiel@uni-hohenheim.de (J.P)
* Correspondence: aelhasan@uni-hohenheim.de; Tel.: +49 711 459 22392 (A.E.)
ralf.voegele@uni-hohenheim.de; Tel.: +49 711 459 22387 (R.T.V.)

Supplementary Material

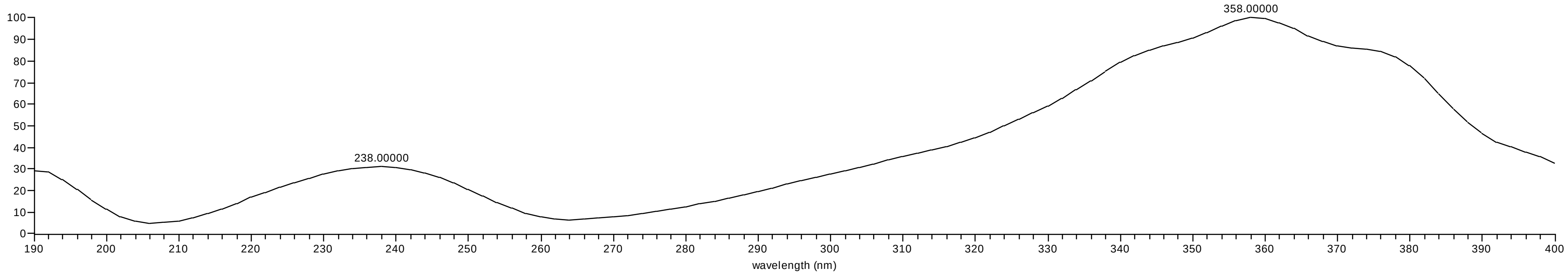

Figure S1. UV-spectrum of peak 1 (retention time of 7.52 min) in fraction F416

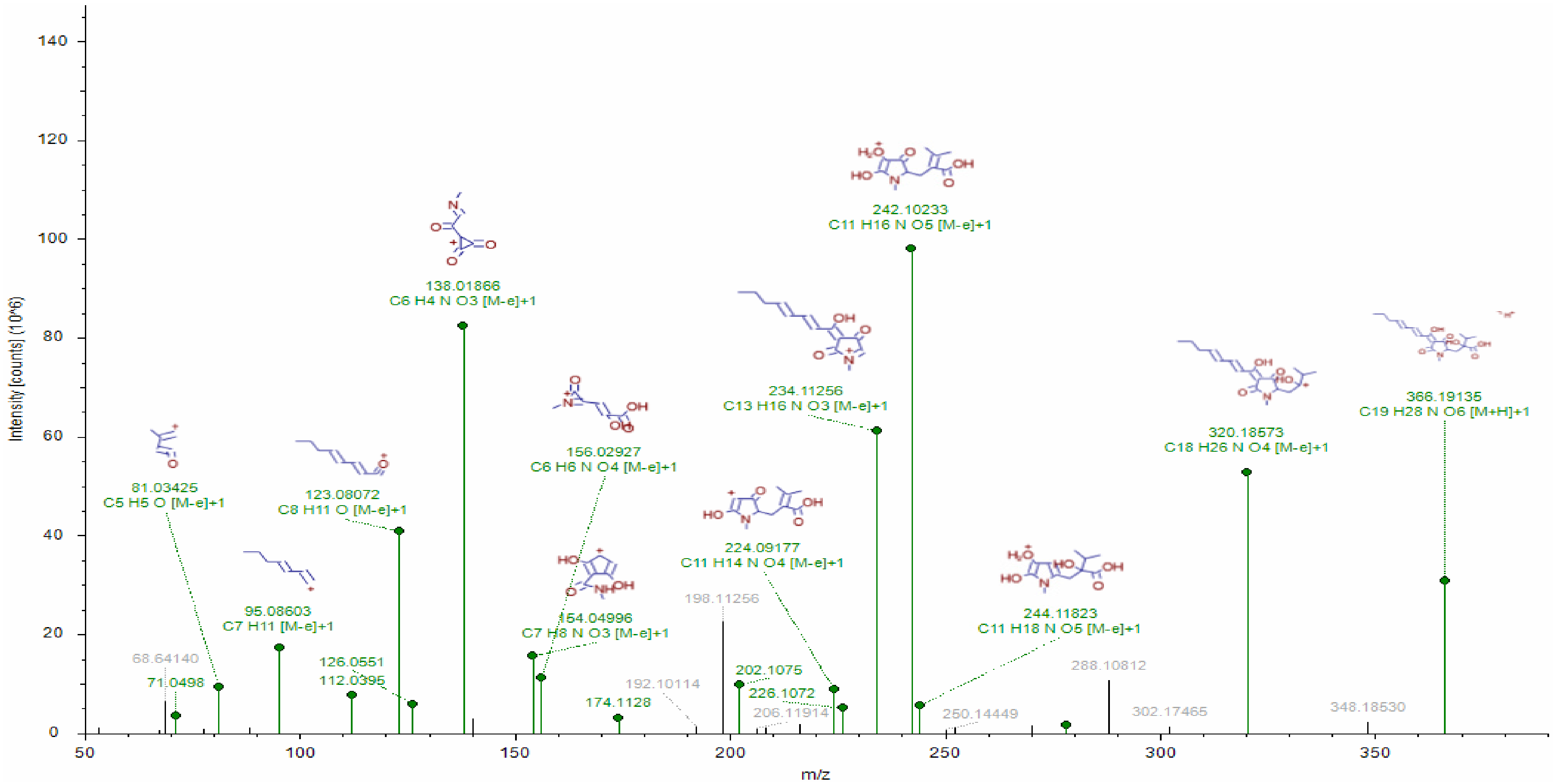

Figure S2. FISh scoring using in silico prediction. MS2 fragmentation spectrum for F416 (harzianic acid) with the precursor ion of m/z 366.19135. The FISh coverage score is 70.4% where 19 product ions were successfully matched (green) and 8 ions remain unmatched. Structural annotations are displayed for all matched ions. The FISh coverage score is determined by the number of matched centroids divided by the number of used (matched and unmatched) centroids multiplied by 100.

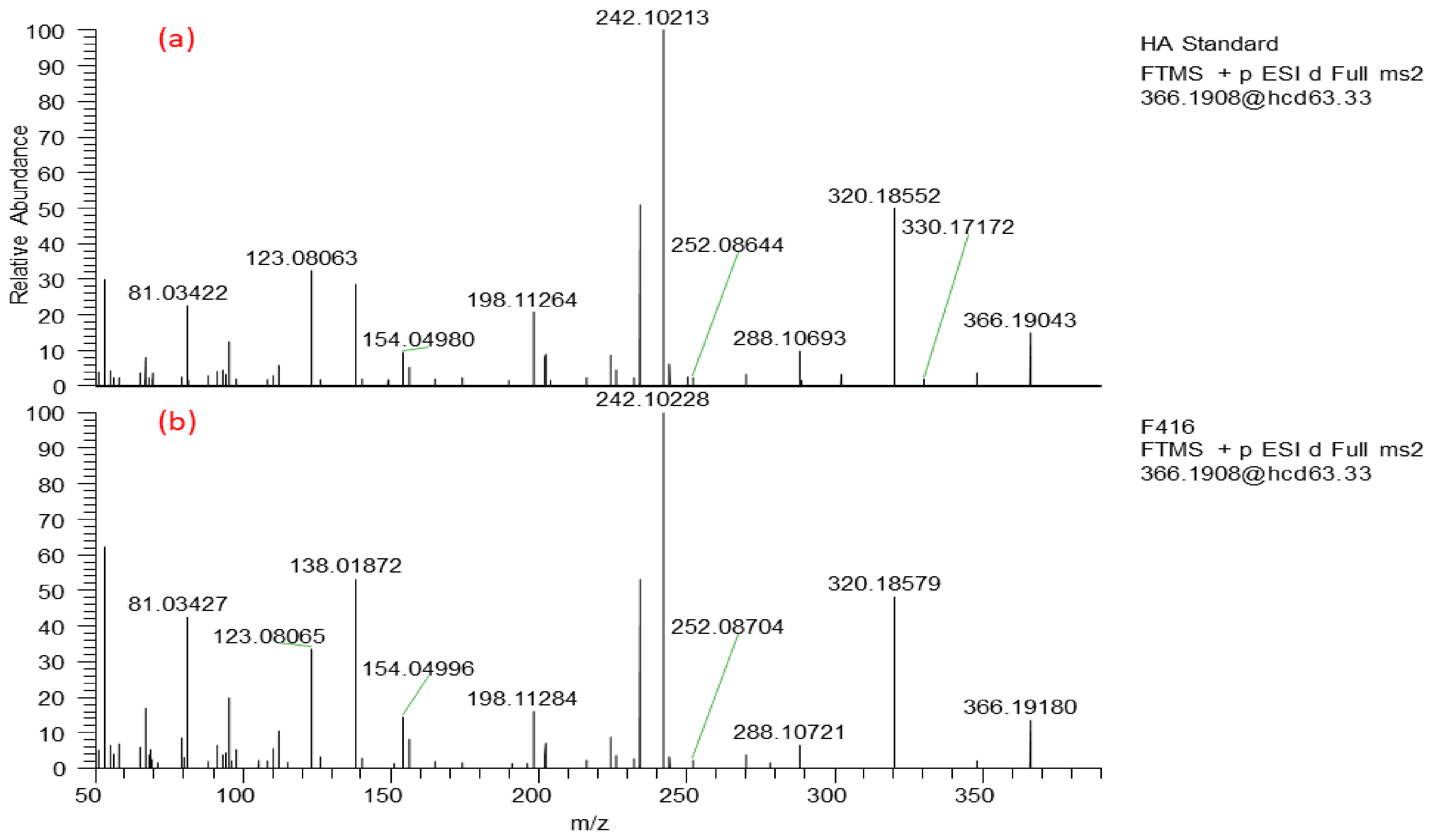

Figure S3. MS2 spectra of HA analytical standard (a) and F416 (b) obtained by LC-ESI-MS/MS analysis in positive ion mode

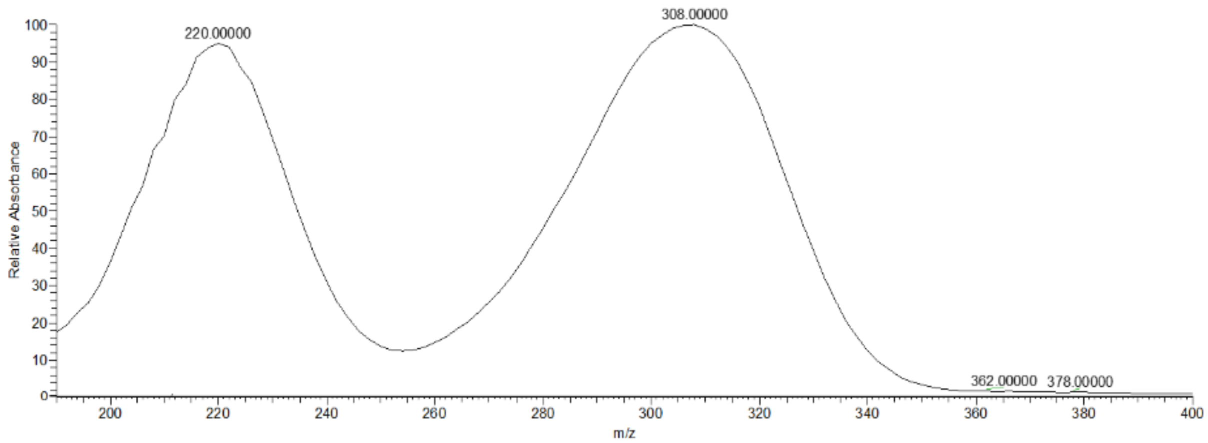

Figure S4. UV-spectrum of peak 3 (with retention time of 5.40 min) in fraction F616

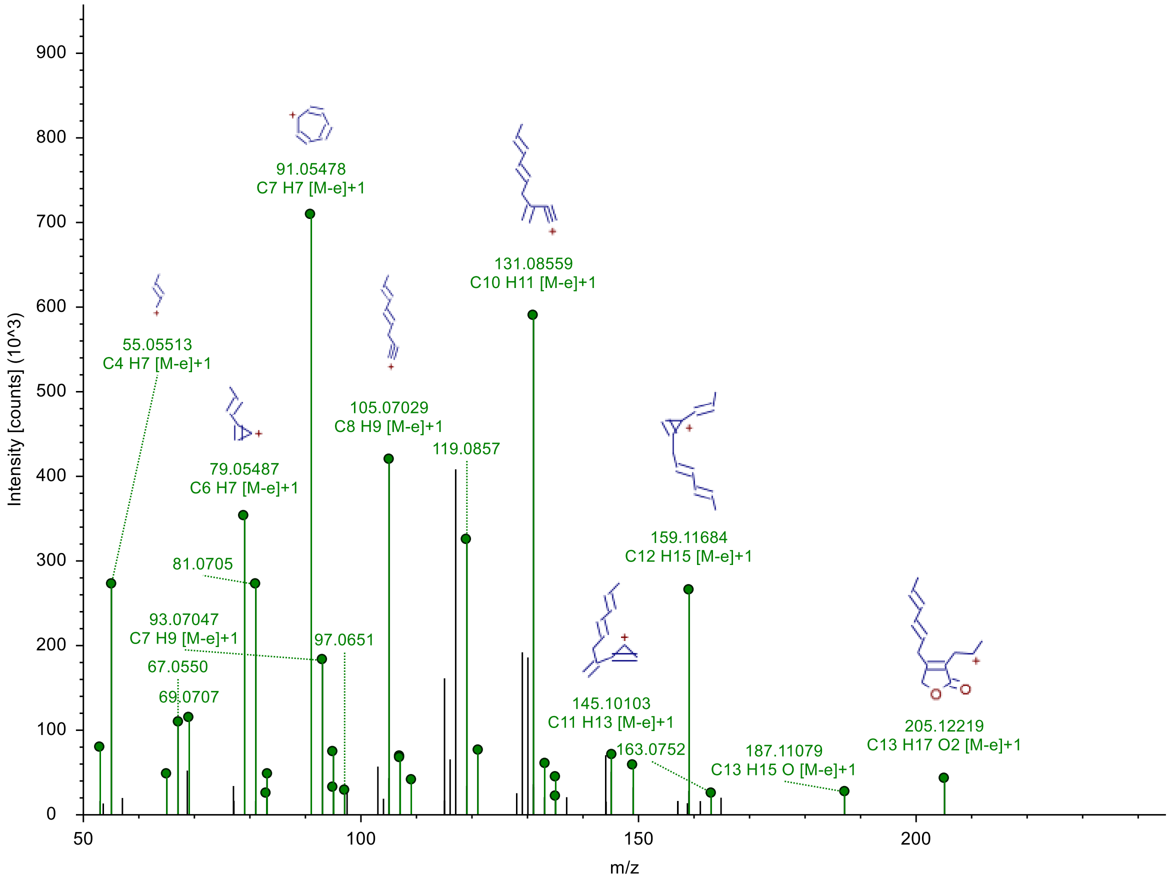

Figure S5. FISh scoring using in silico prediction. MS2 fragmentation spectrum for F616 (harzianolide) with the precursor ion of m/z 223.13249. The FISh coverage score was 62.5 % where 30 product ions were successfully matched (green) and 18 ions remain unmatched. Structural annotations are displayed for all matched ions.

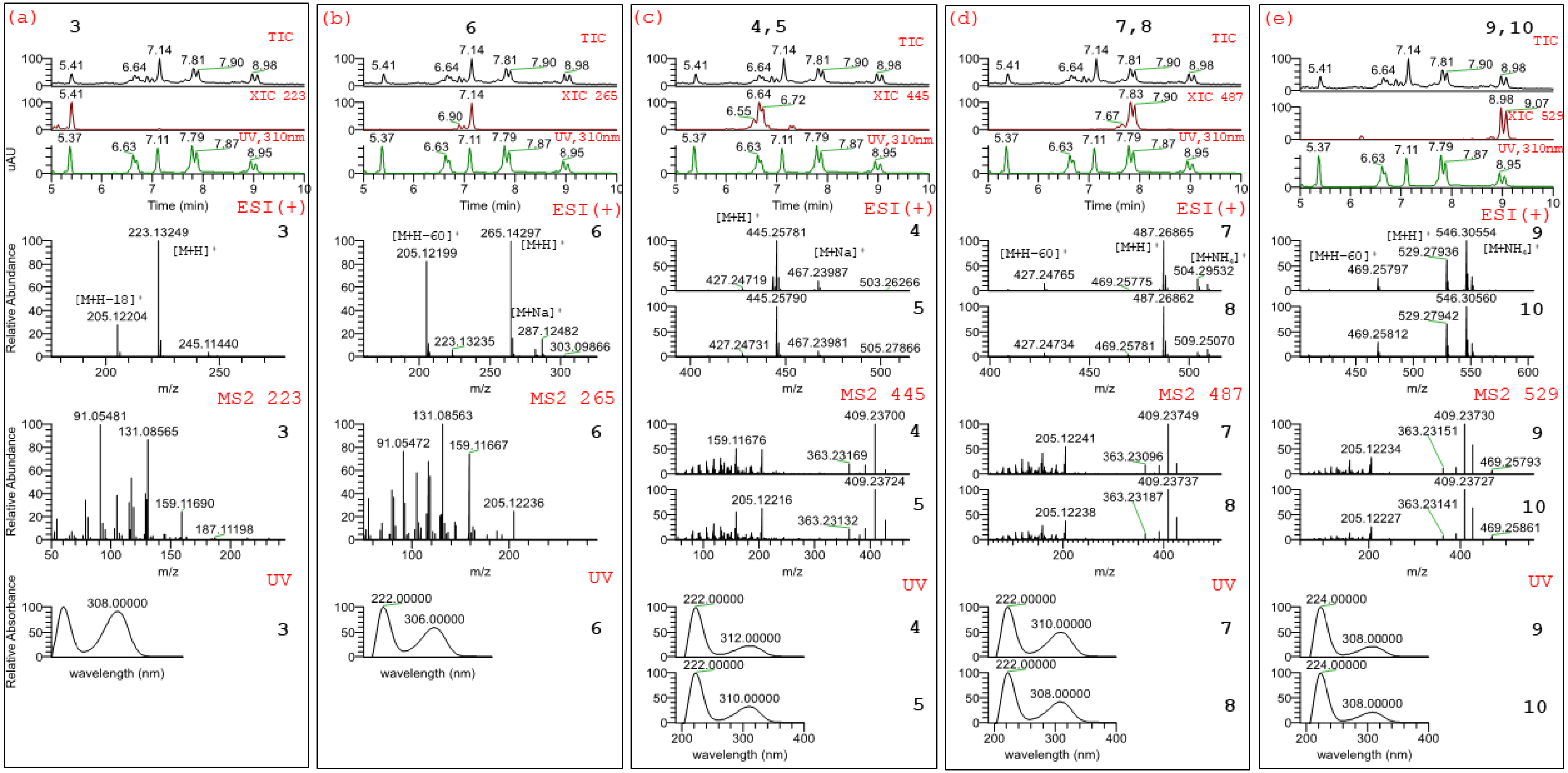

Figure S6. (a): ESI(+)LC/MS/MS data and UV spectra of harzianolide (**3**), (b): ESI(+)LC/MS/MS and UV data of *m/z* 265 (**6**), (c): ESI(+)LC/MS/MS and UV data of *m/z* 445 (**4**,**5**), (d): ESI(+)LC/MS/MS and UV data of *m/z* 487 (**7**,**8**), (e): ESI(+)LC/MS/MS and UV data of *m/z* 529 (**9**,**10**).

Table S1. Mass spectral data of the secondary metabolites released in culture of *T. harzianum* strain T16

Peak	RT [min]	Molecular formula	$[M+H]^+$	Compound	ESI(+)LC/MS/MS
1	7.52	$C_{19}H_{27}NO_6$	366.19040	harzianic acid	320,242,234,138,123,95,81
2	8.40	$C_{19}H_{27}NO_6$	366.19055	iso-harzianic acid	320,270,252,242,234,138,123,95,81
3	5.41	$C_{13}H_{19}O_3$	223.13249	harzianolide	205,159,131,117,91
4	6.63	$C_{26}H_{37}O_6$	445.25781	unknown	427,409,391,363,205,159,131,119,93
5	6.69	$C_{26}H_{37}O_6$	445.25790	unknown	427,409,391,363,205,159,131,119,93
6	7.11	$C_{15}H_{21}O_4$	265.14297	tent. harzianolide acetylated	205,159,131,117,91
7	7.79	$C_{28}H_{39}O_7$	487.26865	unknown	469,427,409,391,363,205,159,131,91
8	7.87	$C_{28}H_{39}O_7$	487.26862	unknown	469,427,409,391,363,205,159,131,91
9	8.95	$C_{30}H_{41}O_8$	529.27936	unknown	469,427,409,391,363,205,159,131,119,91
10	9.05	$C_{30}H_{41}O_8$	529.27942	unknown	469,427,409,391,363,205,159,131,119,91

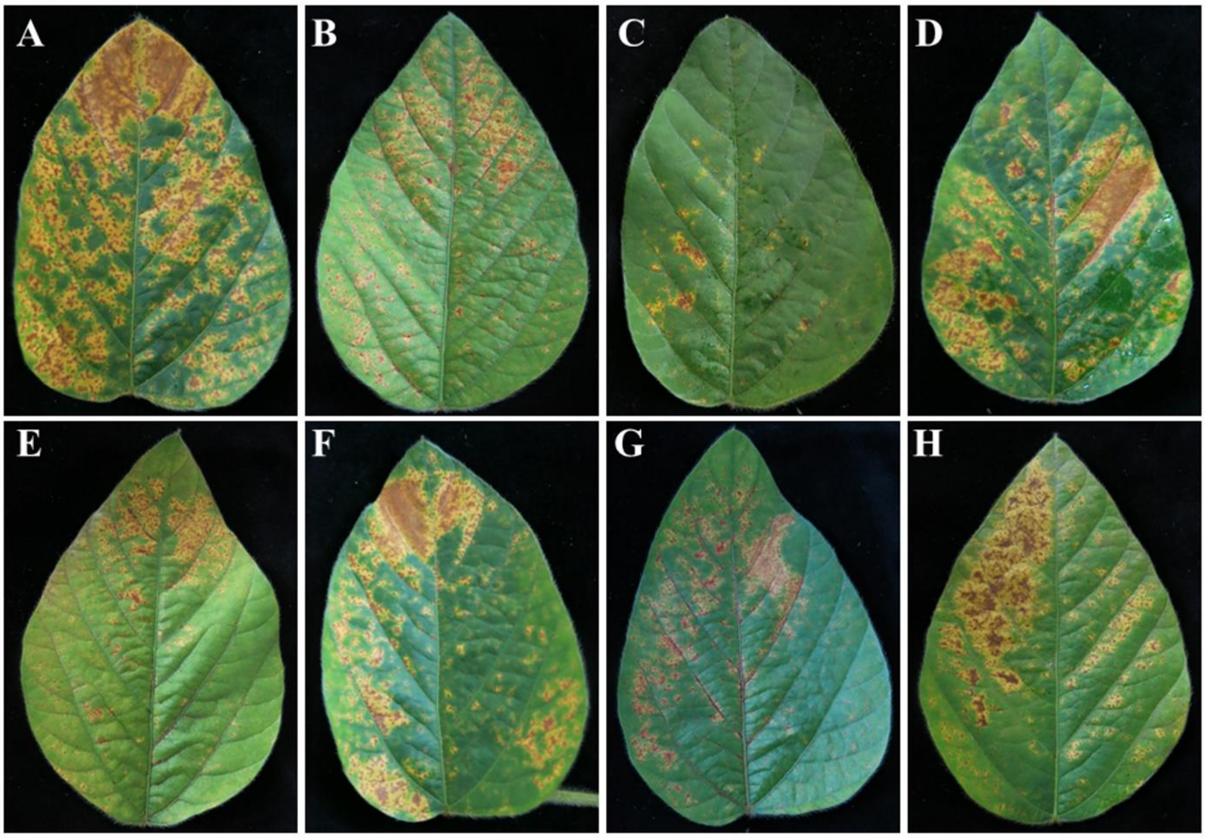

Figure S7. ASR severity on leaves of soybean plants that received on the right side 300 µl 2% acetone (**A**), equal amounts of conidial suspensions (2.5×10^7 conidia mL^{-1}) of T23 (**B**), or T16 (**E**), or metabolites solutions (200 ppm) of 6PAP (**C**), VFA (**D**), F116 (**F**), HA (**G**) and HZL (**H**), 24 h before inoculation with *P. pachyrhizi*.

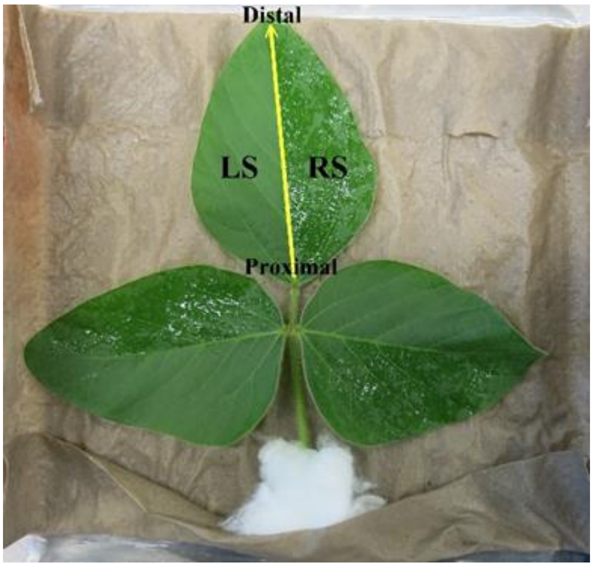

Figure S8. Application of the metabolites or conidial suspensions of *Trichoderma* spp. on the right side (RS) of the adaxial surface of soybean leaves.
